# Crystal Structure of the Hexachlorocyclohexane Dehydrochlorinase (LinA-Type2): Mutational Analysis, Thermostability and Enantioselectivity

**DOI:** 10.1371/journal.pone.0050373

**Published:** 2012-11-27

**Authors:** Ankit S. Macwan, Vandna Kukshal, Nidhi Srivastava, Saleem Javed, Ashwani Kumar, Ravishankar Ramachandran

**Affiliations:** 1 Environmental Biotechnology Division, CSIR-Indian Institute of Toxicology Research, Mahatma Gandhi Marg, Lucknow, India; 2 Department of Biochemistry, Hamdard University, New Delhi, India; 3 Molecular and Structural Biology Division, CSIR-Central Drug Research Institute, Chattar Manzil, Mahatma Gandhi Marg, Lucknow, India; Indian Institute of Science, India

## Abstract

Hexachlorocyclohexane dehydrochlorinase (LinA) mediates dehydrochlorination of γ-HCH to 1, 3, 4, 6-tetrachloro-1,4-cyclohexadiene that constitutes first step of the aerobic degradation pathway. We report the 3.5 Å crystal structure of a thermostable LinA-type2 protein, obtained from a soil metagenome, in the hexagonal space group P6_3_22 with unit cell parameters *a* = *b* = 162.5, *c* = 186.3 Å, respectively. The structure was solved by molecular replacement using the co-ordinates of LinA-type1 that exhibits mesophile-like properties. Structural comparison of LinA-type2 and -type1 proteins suggests that thermostability of LinA-type2 might partly arise due to presence of higher number of ionic interactions, along with 4% increase in the intersubunit buried surface area. Mutational analysis involving the differing residues between the -type1 and -type2 proteins, circular dichroism experiments and functional assays suggest that Q20 and G23 are determinants of stability for LinA-type2. It was earlier reported that LinA-type1 exhibits enantioselectivity for the (−) enantiomer of α-HCH. Contrastingly, we identified that -type2 protein prefers the (+) enantiomer of α-HCH. Structural analysis and molecular docking experiments suggest that changed residues K20Q, L96C and A131G, vicinal to the active site are probably responsible for the altered enantioselectivity of LinA-type2. Overall the study has identified features responsible for the thermostability and enantioselectivity of LinA-type2 that can be exploited for the design of variants for specific biotechnological applications.

## Introduction

**Figure 1:**
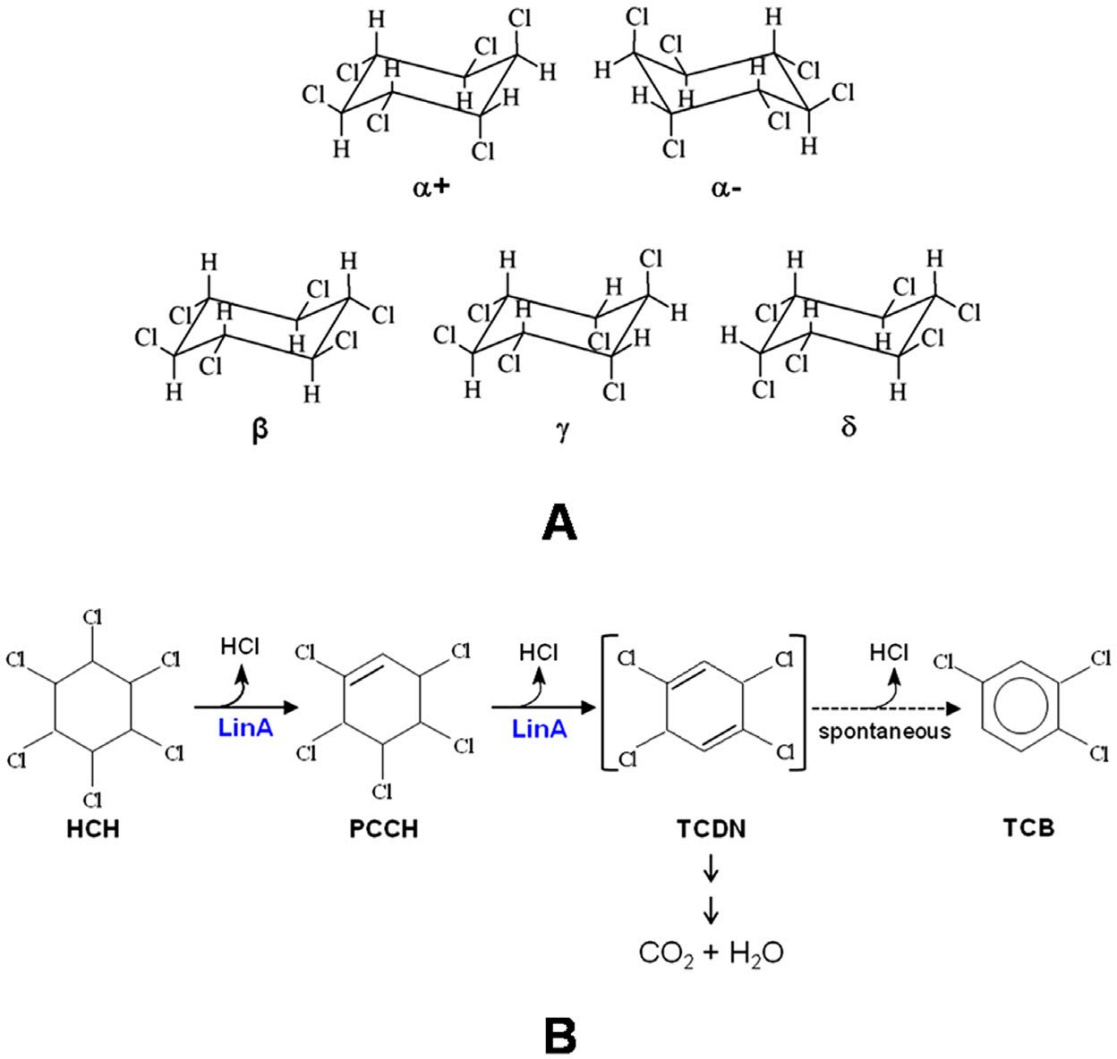
Substrate HCH and generalized reaction of LinA. (A) Enantiomers and isomers of Hexachlorocyclohexane (HCH). (B) Generalized reaction of hexachlorocyclohexane dehydrochlorinase (LinA). LinA converts α-, γ- & δ-HCH to tetrachloro-cyclohexadiene (TCDN) *via* pentachlorocyclohexene (PCCH). The TCDN is metabolized further by other enzymes of the pathway. In the absence of other enzymes TCDN is spontaneously converted to trichlorobenzene (TCB).

Chlorinated insecticide, technical-hexachlorocyclohexane (t-HCH), predominantly consists of four isomers; α-(60–70%), β-(5–12%), γ-(10–15%) and δ-HCH (6–10%) that differs in the spatial distribution of chlorine atoms on the cyclohexane ring (**Figure 1a**). Due to extensive use in the past for protection of crops and control of vector borne diseases, its residues have accumulated at the applied sites and imparts toxicity [1]. Several HCH-degrading microorganisms have been characterized from different parts of world and the pathway for their aerobic degradation has been studied [2], [3].

**Figure 2:**
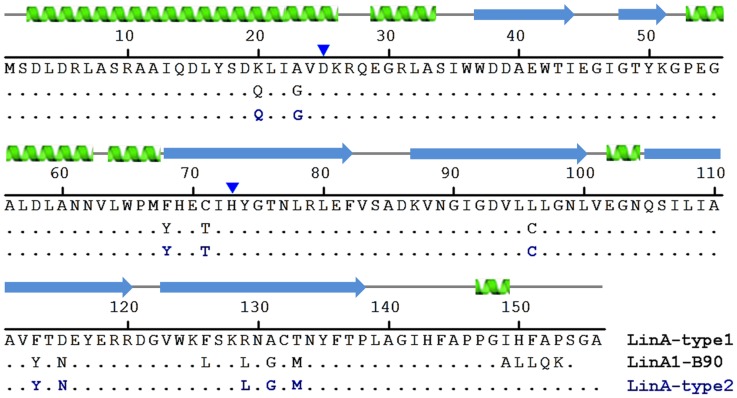
Sequence homology in LinA. The LinA-type1, LinA1-B90 and the -type2 enzyme have been compared. The sequence positions of the catalytic dyad consisting of H73 and D25 have been indicated by *blue* arrows. The secondary structure elements are also indicated.

γ-hexachlorocyclohexane (γ-HCH)-dehydrochlorinase (LinA) mediates the first step of aerobic microbial degradation of γ-HCH to 1,3,4,6-tetrachloro-1,4-cyclohexadiene [4], which is further metabolized by the sequential activity of LinB, LinC, LinD, LinE etc. [2], [3] into substrates that enter the TCA cycle (**Figure 1b**). Additionally, LinA can also use α-, and δ-HCH as substrates but not the β-isomer [5]. The lack of interactions with β-HCH has been attributed to lack of a 1,2-biaxial HCL pair. Several variants of LinA that are >85% identical in sequence have been described [2], [3]. The archetypal LinA-UT26 was characterized from *Sphingobium japonicum* UT26 [2], [4]. It consists of 156 amino acids, and is referred to here as LinA-type1. One of our groups recently described isolation of another variant, LinA-type2, that differs from LinA-type1 by 10 residues [6]. Characterization of yet another variant, LinA1-B90, from *Sphingobium indicum* Β90 has also been described [7]. It consists of 154 amino acids, is 99% identical to the LinΑ-type2 in 1–148aa region. It differs completely in the C-terminal region, where the direct repeat is replaced by ALLQK (**Figure 2**) due to transposition of IS*6100* at 3′ end of its gene [7].

Crystal structure of LinA-type1 has been reported recently [8]. Briefly, it exists as a homotrimer, where each protomer forms a cone-shaped α+β barrel fold. The C-terminal region (residues 139–153) of each protomer extends away from its core structure, interacts with the β6 strand of the neighbouring subunit, and probably provides stability to the trimer by inter-subunit interactions. This conformation is termed as the ‘open’ form as this leaves the substrate cavity accessible. Although a structure of its ‘closed’ form is not known as yet, comparisons with a related enzyme, scytalone dehydratase, suggests that the C-terminal stretch of LinA should cover the substrate cavity in its ‘closed’ form. Inside the barrel fold of each protomer, there is a hydrophobic cavity that includes its active site, and residues K20, I44, L64, V94, L96, I109, A111, F113, A131, C132, and T133 that are involved in interactions with the substrate. Among other things, H73 functions as a base that abstracts the proton of γ-HCH, leading to its dehydrochlorination [8].

LinA variants exhibit considerable differences in their thermostability and enantioselectivity. Thus, while LinA-type2 is thermostable and remains active even after 8 hours of incubation at 45°C, >50% activity of LinA-type1 is lost after incubation for 60 min at the same temperature [6]. Their Tm, analyzed by circular dichroism studies, are 65 and 45°C, respectively. Besides, LinA-type1 and LinA1-B90 variants exhibit preference for transformation of (−) and (+) enantiomers of α-HCH, respectively [9].

Here we show that LinA-type2 exhibits preference for (+) enantiomer of α-HCH and also report its crystal structure. Structural analysis, enzyme activity assays, stability of various mutants and *in silico* docking enabled a rationalization of the thermostability and enantiomer preference of LinA-type2.

## Results

### Crystal Structure of LinA-type2

**Figure 3:**
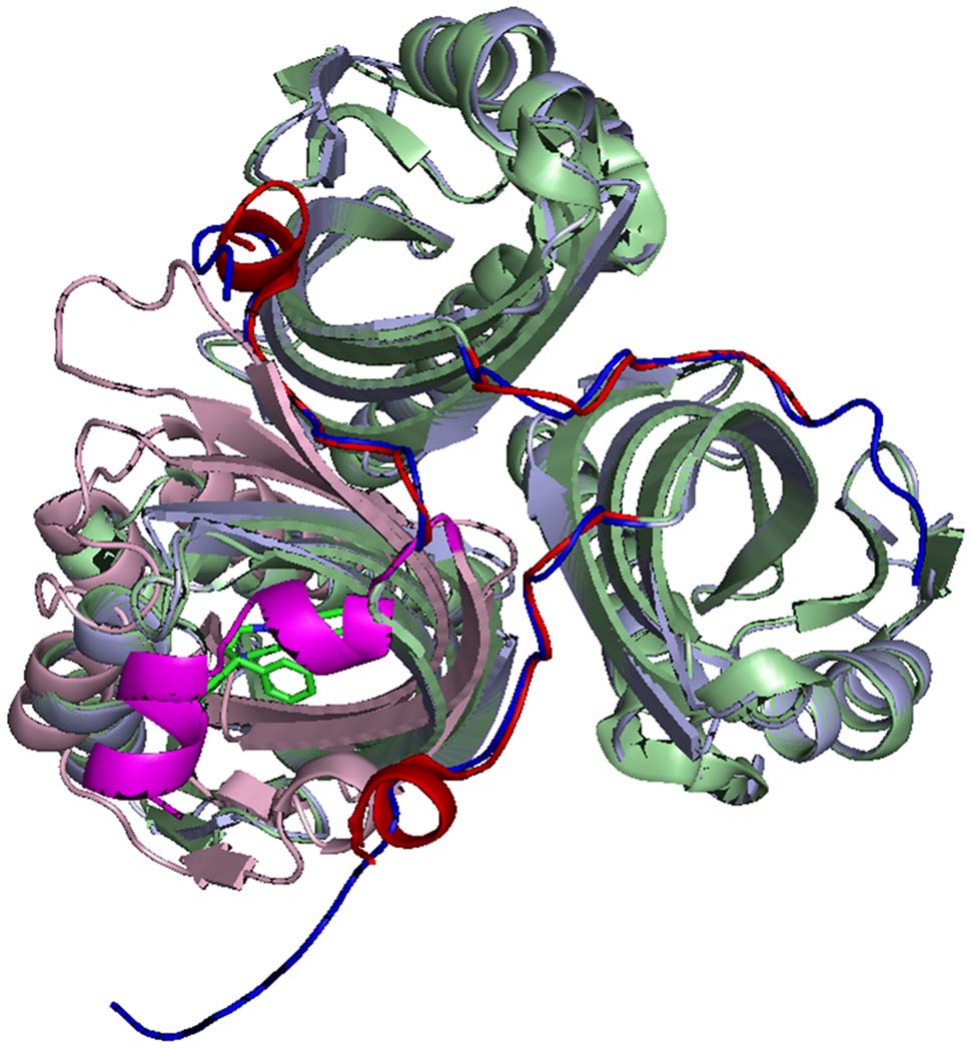
Quaternary association in LinA-type2. LinA-type1 (light green) and scytalone dehydratase (PDB: 3STD, light pink) are shown superposed onto the -type2 trimer. The C-terminal segment is clearly mobile and its spatial dispositions in the -type2 structure are consistent with the ‘open’ conformation of the protein where the substrate access to the active site is not hindered. The C-terminal region (magenta) of scytalone dehydratase that is in the closed form and covers the active site cavity is highlighted for clarity.

Crystals of LinA-type2 that diffracted to a moderate resolution of 3.5 Å in the space group P6_3_22 were obtained (**Figure S1**). Molecular replacement studies revealed that the crystal has seven subunits in the asymmetric unit. Three of the subunits associate as a trimer while other protomers form trimers through crystal symmetry (**Figure 3**). The trimeric quaternary association is consistent with size exclusion chromatography involving -type2 protein (*data not shown*) and also with the reported LinA-type1 structure [8].

**Figure 4:**
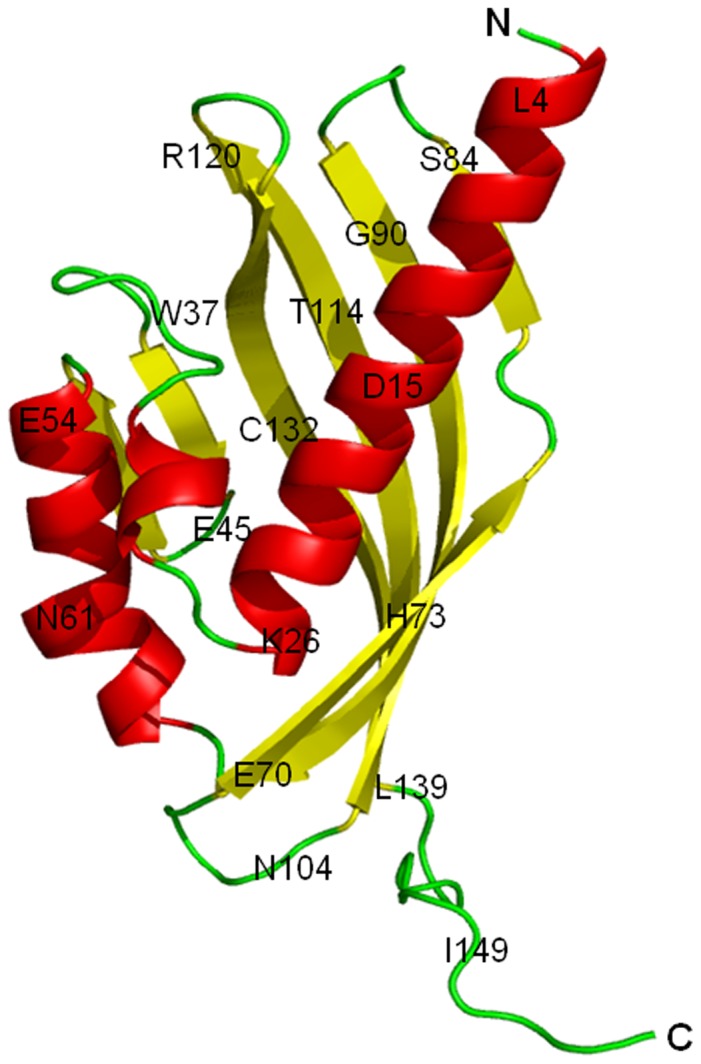
Tertiary structure of LinA-type2. Cartoon representation of the protein with selected residues labeled for clarity. Helices and sheets are depicted in *red* and *yellow*, respectively, while the loops are indicated in *green*.

**Figure 5:**
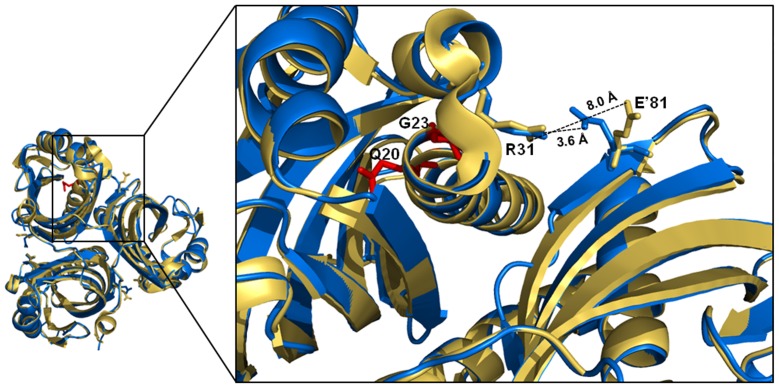
Salt-bridge between R31’-E81 in LinA-type2. A close-up of the LinA-type1 structure superposed onto the -type2 co-ordinates to illustrate the conformational difference between the R31 and E81 residues. The -type1 protein is shown in yellow while the -type2 protein is shown in blue. G23 and Q20, two residues that are important for thermostability of the -type2 protein are depicted as red sticks for clarity.

The broad features of the tertiary structure (**Figure 4**) are very similar to that of the reported -type1 protein [8]. This is not unexpected given that the respective sequences are >92% identical (**Figure 2**). Hence only a short description is given here with emphasis on the differences. Briefly, each protomer consists of six β-strands; residues 36–44 (β1), 48–51 (β2), 68–82 (β3), 87–100 (β4), 105–120 (β5), 123–138 (β6), and four α-helices; residues 3–26 (α1), 29–33 (α2), 52–62 (α3) and 64–67 (α4), forming a cone-shaped α+β barrel fold. The root mean square deviation between the Cα atoms of the protomers in the -type2 enzyme varies between 0.34 and 0.52 Å, while it varies between 0.38 and 0.64 Å compared to -type1. Small differences occur mainly in C-terminal segment of various protomers in the asymmetric unit, and also with -type1 protein, suggesting that C-terminal segment of LinA-type2 is quite mobile.

**Table 1:** Intersubunit salt-bridges in both LinA proteins.

	LinA-type2			LinA-UT26		
No	Monomer A	Dist. [Å]	Monomer E	Monomer C	Dist. [Å]	Monomer B
1	ASP 15 [OD2]	3.0	ARG 10 [NH1]	ASP 15 [OD2]	3.3	ARG 10 [NH1]
2	ASP 15 [OD1]	2.6	ARG 10 [NH2]	ASP 15 [OD1]	3.6	ARG 10 [NH2]
3	ASP 15 [OD2]	3.6	ARG 79 [NE ]	ASP 15 [OD1]	3.5	ARG 79 [NE ]
4	ASP 19 [OD2]	3.1	ARG 79 [NH1]	ASP 19 [OD1]	3.0	ARG 79 [NH1]
5	**ASP 19 [OD1]**	**2.5**	**ARG 79 [NH1]**	**ASP 19 [OD2]**	**2.4**	**ARG 79 [NH1]**
6	**LYS 26 [NZ ]**	**2.5**	**ASP 93 [OD1]**	**LYS 26 [NZ ]**	**2.7**	**ASP 93 [OD1]**
7	LYS 26 [NZ ]	3.3	ASP 93 [OD2]	LYS 26 [NZ ]	3.5	ASP 93 [OD2]
8*	ARG 31 [NH2]	3.6	GLU 81 [OE1]			

Potential salt-bridges with distance cut-off of 3.6 Å between respective atoms. Salt bridges reported in the earlier LinA-UT26 are shown in bold.

* Additional salt bridge identified in the -type2 protein. Calculations were performed using PISA (http://www.ebi.ac.uk/msd-srv/prot_int/pistart.html).

**Table 2:** Comparison of intersubunit buried surface area in both LinA types.

	Interfacing structures	Buried area, Å2 (%)
**LinA-type1**	C+B	1078.3 (13%)
	B+A	1063.5 (13%)
	C+A	1009.0 (13%)
	Average	**1050.3 (13%)**
**LinA-type2**	E+A	1163.2 (17%)
	C+A	1095.5 (16%)
	E+C	1087.9 (16%)
	Average	**1115.5 (17%)**

Calculations were carried out using PISA (http://www.ebi.ac.uk/msd-srv/prot_int/pistart.html).

The C-terminal segment has been implicated in the catalytic mechanism of LinA proteins, where based on its spatial position, the enzyme is thought to be in an ‘open’ or ‘closed’ form [8]. The reported structure of LinA-type1 was identified to be in the ‘open’ form as, in contrast with the related enzyme scytalone dehydratase [10], the C-terminal segment did not cover the hydrophobic cavity formed by the α+β barrel fold. Superimposition of LinA-type2 with these structures (**Figure 3**) revealed that the crystal structure reported in the present report is consistent with ‘open’ form of the enzyme.

**Figure 6:**
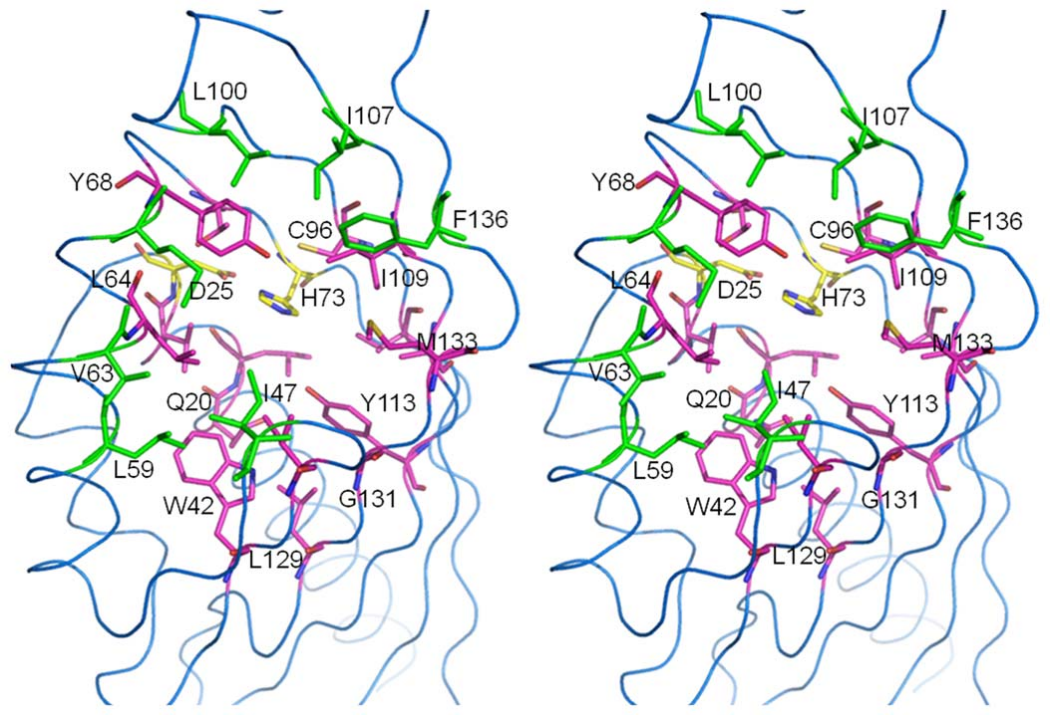
Active site of LinA-type2. Stereo representation of the active site of LinA-type2. The protein is depicted as a blue ribbon while residues that straddle the entrance to the active site are shown as green sticks and labeled for clarity. Pink residues show those that are inside the active site pocket while the H73 and D25 that form the active site ‘dyad’ are shown in yellow stick representation.

**Figure 7:**
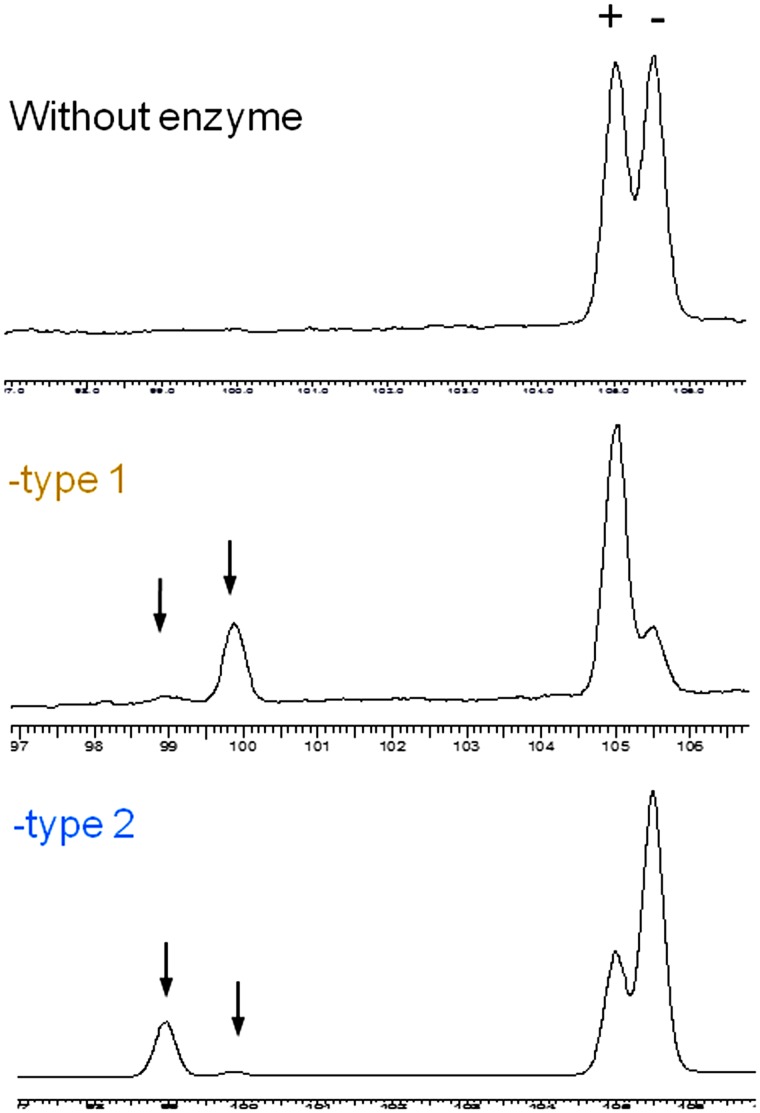
Enantioselectivity of LinA-type2. Enantioselective transformation of (+) and (−) α-HCH by LinA-type1 & -type2 proteins respectively, analyzed using chiral capillary column gas chromatography as detailed in *Materials & methods*. The arrows indicate the position of the pentachlorocyclohexene products. Clearly the -type1 and -type2 proteins exhibit opposite enantiomer specificity.

**Figure 8:**
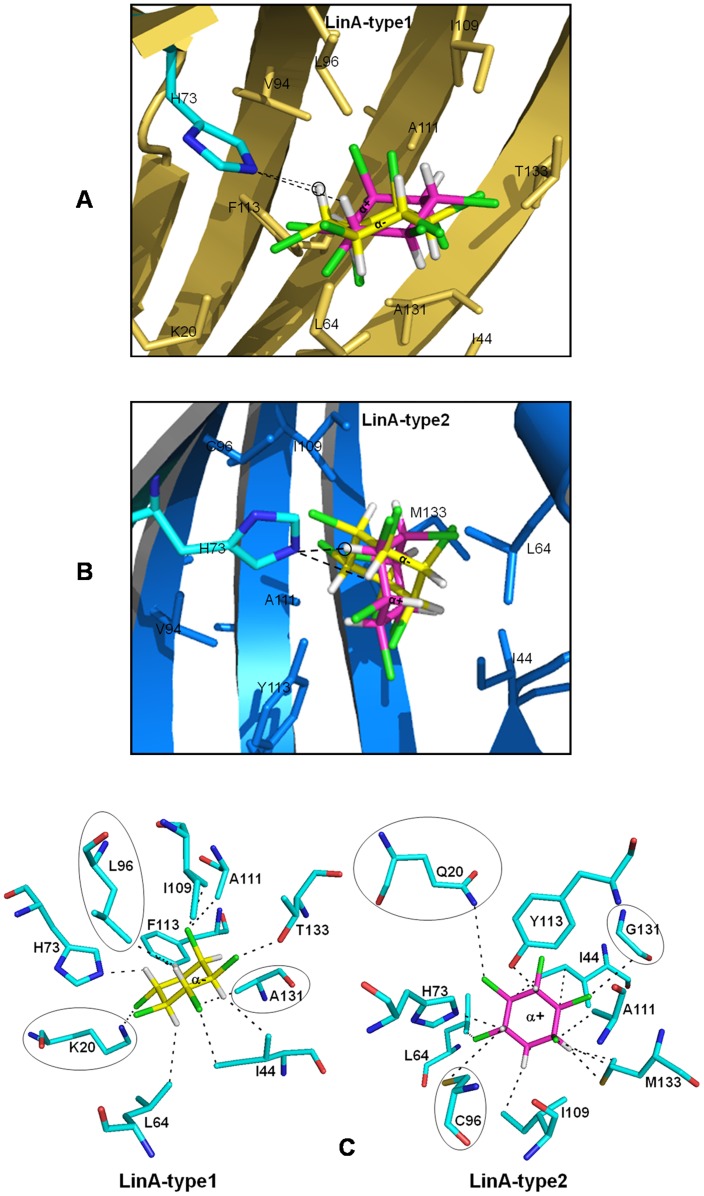
*In silico* docking of the enantiomers of the α-HCH substrate. Docking of the substrates against the -type1 protein (panel A) shown in yellow cartoon representation and against the -type2 protein (panel B) shown in blue. Residues that are vicinal to the substrate (≤3.5 Å) are indicated. Interactions of the nitrogen atom of the catalytic H73 to the proton of (+)- or (−)-α-HCH is indicated by dashed lines with the closer hydrogen atom shown encircled. (C) Enantioselectivity in LinA proteins. The interactions of the (−) enantiomer of α-HCH (yellow) with the -type1 protein are depicted in the left panel. The right panel shows the interactions of the -type2 protein with the (+) enantiomer of the substrate shown in magenta. The comparison shows that the interactions of residues K20, L96 & A131 (shown encircled) with (−) α-HCH in the -type1 protein is lost upon the change to Q20, C96 & G131 residues, respectively, in the -type2 protein.

**Table 3:** Relative activity of LinA-type2 mutants.

No	LinA-	Amino acid position										Relative Activity (%)
		20	23	68	71	96	113	115	129	131	133	
1	type1	K	A	F	C	L	F	D	R	A	T	95
2	type2	Q	G	Y	T	C	Y	N	L	G	M	100
3	M1	o	o	Y	T	C	Y	N	L	G	M	44
4	M2	o	o	o	o	C	Y	N	L	G	M	44
5*	M3	o	o	o	o	o	Y	N	L	G	M	–
6*	M4	o	o	o	o	o	o	o	L	G	M	–
7	M5	Q	G	Y	T	C	Y	N	O	o	o	62
8	M6	Q	G	Y	T	C	o	o	O	o	o	33
9	M7	Q	G	Y	T	o	o	o	O	o	o	58
10	M8	Q	G	o	o	o	o	o	O	o	o	77
11	M9	Q	o	o	o	o	o	o	O	o	o	60
12	M10	o	G	o	o	o	o	o	O	o	o	65

The respective relative activities were directly determined at 30°C using the α-HCH as the substrate.

The activity of LinA-type2 was taken to be 100%.

Dots are put to show that the respective residues are conserved between the -type1 protein and the respective constructs.

* Could not be determined as the respective mutant proteins were not present in the soluble fraction.

Quaternary association of LinA-type2 is stabilized by several salt bridges (**Table 1**). While some of these like K26-D93’ and D19-R79’ were identified earlier in LinA-type1 and are also conserved in scytalone dehydratase [10], a new salt bridge, was found between R31-E81’ in LinA-type2 (**Figure 5** **and also Figure S2**). The average B-factors of these residues were found to range between 29.1–83.2 Å^2^ for R31 and between 32.2–71.7 Å^2^ for E81 in the seven individual chains present in the asymmetric unit. The respective values are in close agreement with the average B-factors of the individual protein chains. The overall average B-factor for R31 and E81 is 49.09 and 52.25 Å^2^, respectively, while that for the structure as a whole is 50.19 Å^2^. Intersubunit buried surface area analysis showed that 17% of the accessible surface area is buried in -type2 protein with an overall average of 1115.5 Å^2^ (**Table 2**). This is about 4% less in -type1 protein where the average intersubunit buried surface area is 1050.3 Å^2^.

**Figure 9:**
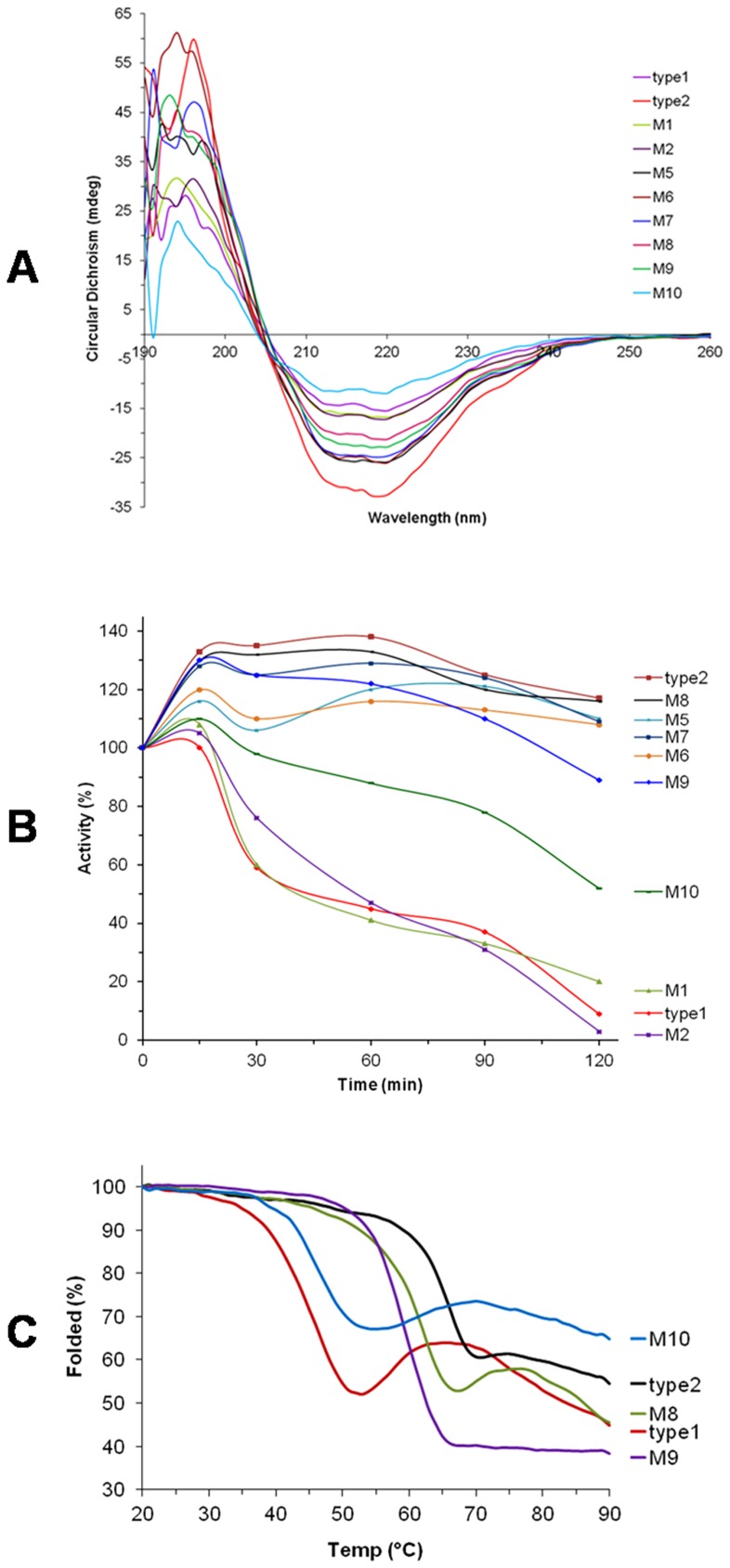
Far UV CD, relative activity and thermal unfolding of the proteins. (A) Far UV CD scans between 190–260 nm of the type-1, -type2 and mutant LinA proteins show that the mutants are well-folded comparable to the corresponding native proteins (B) The relative activity (%) is shown plotted against time (min). The mutants correspond to those detailed in Table3. Residual α-HCH dehydrochlorinase activity was measured after pre-incubation of LinA proteins at 60°C for different time periods. Activities prior to pre-incubation were taken as 100%. (C) Thermal unfolding of the proteins followed by Circular dichroism spectroscopy. Clearly mutants that contain Q20 and G23 exhibit higher thermostability comparable to that of the native -type2 protein.

**Table 4:** Data collection and refinement statistics for LinA-type2.

Data collection statistics			
Wavelength (Å)			1.5418
Space group			P6_3_22
Cell dimensions: *a, b, c* (Å)			107.5, 107.5, 52.3
Resolution range (Å)			30.72−3.5 (3.59−3.5)*
No. measured reflections			89702 (12204)
No. unique reflections			18690 (2656)
Multiplicity			4.8 (4.6)
I/σ(I)			8.7 (2.7)
Completeness (%)			99.1 (98.5)
R_merge_			0.239 (0.663)
**Refinement statistics**			
Protein atoms			8266
R_factor_ (%)			17.9
R_free_ (%)			27.1
Mean B-factor (Å^2^)		Main chain	49.9
		Side chain	50.4
R.m.s.d. bond lengths (Å )			0.011
R.m.s.d. bond angles (°)			1.445
Ramachandran plot	Most favored regions (%)		91.6%
	Additionally allowed regions (%)		7.4%

* Values in parentheses are for the highest resolution shell (3.59−3.5 Å).

R_merge_ = ΣhΣi|Ih, i−Ih|/ΣhΣiIh,i, where Ih is the mean intensity of the i observations of symmetry related reflections of h. R_factor_ = Σ|Fobs−Fcalc|/ΣFobs, where Fobs = FP, and Fcalc is the calculated protein structure factor from the atomic model. R_free_ was calculated using 5.0% of the data that was not used during the refinements.

### Description of the Active Site

The active site of LinA is largely hydrophobic in character and is located in the characteristic cavity of the tertiary barrel fold. It is formed by the β-strands at one end and the α-helices at the other (**Figure** **6**). The maximum number of residue changes between the -type1 and -type2 proteins occurs at the active site. On the other hand, a structure-based comparison with the earlier -type1 structure suggests that the entrance to the active site consists of many hydrophobic residues that are mostly conserved between LinA proteins. The conserved residues include I44, I47, L59, V63, M67, L100, I107, and F136. The main differences T133M, F68Y and A131G, present vicinal to the entrance to the active site in -type2 protein might lead to overall reduced substrate accessibility. Incidentally T133 that is now M was suggested to be important for interactions with the HCH moiety in -type1 protein [8]. Analysis of the active site residues itself shows that the -type2 protein exhibits several differences including K20Q, F68Y, C71T, L96C F113Y, R129L and A131G, compared to -type1 protein. The other residues, *viz*., L21, V24, D25, W42, I44, L64, H73, V94, L96, I109 and A111 are conserved between the -type1 and -type2 proteins (**Figure 2**). H73 and D25 are known to be the catalytic ‘dyad’ that is essential for the activity, however, is conserved between the LinA proteins.

### Enantioselectivity of LinA-type2 and Structure Based Rationalization

In contrast with the preferential transformation of α(−) HCH by LinA-type1 [9], LinA-type2 exhibited preference for α(+) HCH (**Figure 7**). As our attempts to co-crystallize LinA-type2 with its substrate were unsuccessful, we carried out *in silico* docking analysis to understand how LinA-type2 is able to distinguish between the α(+) & α(−) HCH enantiomers. Analysis for both LinAs with α(+) or α(−)-HCH as ligand, using GOLD suite of programs, showed that α-HCH moiety was bound to the same region as reported earlier for -type1 protein and γ-HCH [8] (**Figure 8**). *Goldscore* values of 38.45 and 23.96 respectively for the α(+) & α(−) HCH-LinA-type2 complexes, respectively (**Figure 8a**), agree with the experimental results that the -type2 protein should prefer α(+) HCH. Corresponding conclusions for the -type1 protein based on the *Goldscore* values alone was not possible because the values were similar (∼36.05) for both enantiomers (**Figure 8b**).

Further analysis of the active site and interactions of the substrates in the respective docked complexes were carried out. It was identified that residue changes K20Q, F68Y, C71T, L96C, F113Y, A131G and T133M in -type2 are vicinal to the docked HCH moiety and could conceivably alter the contours of the active site to enforce enantioselectivity. Indeed, in the respective complexes, the orientation and distance of the substrates were such that NE2 of H73, important for enzyme activity by abstraction of the proton [8], can interact better with α(−) enantiomer in -type1 protein (**Figure 8c**). Conversely, the same residue is better positioned to interact with α(+) enantiomer in the complex with -type2 protein. Separately, residue F113 that has been proposed to be involved in protein-substrate interactions in -type1 protein [8] is changed to Y in the -type2 protein, and can affect the activity.

### Mutational Analysis of LinA Proteins and Thermostability

Analysis of enzyme activities of a series of mutants, containing one or more of the ten residues that differ between LinA-type1 &-type2 (**Table 3**) suggested the underlying roles of the respective different residues in the activity of the enzyme. The activities of LinA-type1 and -type2 for transformation of α-HCH were comparable, as reported earlier also [6], but activities of all the studied mutants were comparatively lesser. Interestingly the activities of mutants K20Q or A23G were decreased by around 40%, but in the double mutant, K20Q/A23G, a reduction of ∼20% only was detected (**Table** **3**). This suggests that some of the defects of the single mutants are overcome by the double mutant.

Thermostability of LinAs, as measured by loss of activity after incubation at 60°C for different time periods, revealed that while >50% activity of LinA-type1 was lost after one hour, activity of LinA-type2 continued to remain unchanged even after two hours, as reported earlier also [6]. All the studied mutants were properly folded, as measured by Far-UV CD spectra (**Figure** **9a**). Interestingly, the mutants of LinA-type2 that had changes in N-terminal half of the protein conferred thermostability to the protein but those in C-terminal half were ineffective (**Figure** **9b**). Analysis of mutants that had progressively lesser changes in N-terminal half suggested that changes to mainly two residues, *viz*. K20Q and A23G, could confer thermostability to LinA-type2 as compared to the -type1 protein. Further analysis involving single mutants K20Q and (or) A23G respectively suggests that these alone are also sufficient to confer higher thermostability, as compared to the -type1 protein. Circular dichroism spectroscopy experiments (**Figure** **9c**) at progressively higher temperatures revealed that the respective Tm of LinA-type2 and -type1 was ∼65 and ∼45°C, as reported earlier also [6]. The Tm of the mutants M8 (Q20+G23) and M9 (Q20) were comparable to LinA-type2. Tm of mutant M10 (G23) was lesser than -type2 but was still higher than LinA-type1.

## Discussion

Thermostability and enantioselectivity of enzymes are extremely useful for their biotechnological applications. Thus, Thermostable proteins can withstand higher temperatures (>60°C), cause faster rates, better solubility of substrate and decreased side-contamination by microorganisms, among others [11], [12], [13]. Additionally, these can withstand many other harsh conditions that are usually encountered in industry *viz*. extreme pH, high salt, organic solvents etc [14]. Similarly, enantioselectivity of enzymes help in synthesis of the desired enantiomer, while avoiding formation of other enantiomer, which can not only be inactive but can be toxic [15], [16]. The present study was aimed at understanding the features responsible for thermostability and enantioselectivity of LinA proteins.

The structure of the -type2 protein is understandably highly conserved compared to the -type1 protein and therefore it was unanticipated that it would exhibit different enantiomer specificity for α-HCH compared to the -type1 protein. A closer look reveals that changes to the sequence occur mostly around the active site. The analysis of the docked complexes involving both LinA-types and the (+) and (−) enantiomers of α-HCH was therefore carried out. The orientation of α-HCH enantiomers in the pocket of LinA was identified as important in the exhibited preference of the enzyme for one over the other. Thus under favorable substrate binding conditions, the orientation of α(+)-HCH is closer to NE2 of His73, so that it can easily abstract the proton [8] in -type2. Similarly α(−) binds closer to H73 compare to α(+) in -type1. Further the docking results indicate that the changes K20Q, L96C and A131G may be important in altering the enantioselectivity of LinA-type2.

The structural work seems to agree with the experimental results involving activity and stability of LinA-type2; however, the moderate resolution of the crystal structure raises the possibility that some of the detailed analysis may have to be reworked when better diffracting crystals become available. On the other hand, the presence of 7 independent subunits in the asymmetric unit and also the availability of a closely related and structurally well-conserved -type1 protein model gave us confidence in the general rationalization of the results. Salt bridges have been identified amongst the principal determinants for conferring thermostability to proteins [17], [18]. Additionally, inter-subunit buried surface area is important for the stability of multimeric proteins [19]. Presence of an additional salt bridge Arg31’-Glu81 in LinA-type2, and also an increased intersubunit buried area by about 4% can, therefore, could be contributing factors for the observed thermostability of LinA-type2. These residues are also present in LinA-type1but fail to form a bridge, possibly due to steric restrictions by the side chains of changed amino acids in that protein. Amongst the ten residues that differ between -type1 and -type2 proteins, two residues *viz*. Q20 and G23 are important determinants for this property as seen by measurement of enzyme activity and CD-spectroscopy experiments at varying temperatures of a series of LinA-type2 mutants. Similar change in few residues has been shown earlier to be sufficient for conferring thermostability to several other mesophilic proteins as well [20].

Overall the present study has unveiled molecular features responsible for the better thermostability and enantioselectivity of LinA-type2 that is potentially useful in future enzyme engineering for bioremediation and other industrial applications.

## Materials and Methods

### Cloning, Sequencing, Expression and Purification of LinA Proteins

Genes for LinA-type1 (EU863865), and -type2 (EU863871), obtained earlier by PCR from metagenomic DNA of a HCH-contaminated soil [6], were amplified using primer set F (CATATGAGTGATCTAGACAGACTTGCAA) and R (CTCGAGTGCGCCGGACGGTGCGA). After digesting with NdeI and XhoI the products were ligated with NdeI/XhoI digested pET-26b (+) vector (Novagen, Darmstadt, Germany) and cloned in *E.coli* BL21 (DE3). The recombinants were grown in LB-medium and when the growth reached an OD_600_, of ∼0.6, the cells were induced with 0.1 mM IPTG, and harvested after 10 h incubation at 22°C. After a washing step with 50 mM sodium phosphate buffer (pH 8.0), they were sonicated (Ultrasonic processor UP100H, Hielscher, Stuttgart, Germany). After centrifugation at 20,000 g for 30 min, the expressed proteins present in the clear supernatant were purified by Ni-NTA Superflow (QIAGEN, Hilden, Germany) columns at 4°C. Size exclusion chromatography experiments were carried out on an AKTA FPLC (GE Healthcare, Piscataway, NJ, USA), using SuperdexTM200, 10/300 GL column. 50 mM potassium phosphate buffer (pH 8.0) was used as both pre-equilibration and run buffer. Protein estimation was done using the ‘Bio-Rad Protein Assay Reagent’ (Bio-Rad, Hercules, CA, USA) using bovine serum albumin (Sigma, USA) as standard. The purified proteins were diluted to 1.0 mg ml-1 in 50 mM sodium phosphate buffer (pH 8.0) containing 15% glycerol, and stored at−20°C in aliquots.

### HCH-dehydrochlorinase Activity

Activity of respective LinA proteins was determined by following the disappearance of the substrate, as described earlier [6], [21]. Briefly, the reaction medium (1 ml) contained 50 mM Tris-HCl; pH 8.0, 34 µM HCH-isomer (stock solution 1 mg ml-1 in DMSO), 10% glycerol and 10 µg of LinA proteins. Reaction vials in triplicates were set up for each time point, which were withdrawn after incubation at 30°C for different time intervals. The reaction was stopped by acidification to pH<2. Residual HCH was extracted thrice with 1 ml n-hexane and analyzed by gas chromatography [22].

### Thermostability of LinA Proteins

The thermostability of LinA proteins was initially monitored by following the loss of enzyme activity by pre-incubation at 60°C. 1 ml reaction medium that contained 50 mM Tris HCl; pH 8.0, 10% glycerol and 10 µg of either LinA was incubated at 60°C for different time intervals. This was followed by the initiation of reaction by addition of 10 µg α-HCH. The reaction was terminated after further incubation for 10 min at the same temperature by acidification to pH<2.0 and the residual α-HCH was analyzed as above.

Circular Dichroism experiments were also used to probe the thermostability. CD measurements were made using a ChirascanTM Spectrometer (Applied Photophysics, Surrey, United Kingdom) that was calibrated with ammonium (+)-10- camphorsulfonate at 25°C with cell of 1 mm path length. Far-UV spectra scans for the proteins were carried out from 190–260 nm. The values were obtained by using 10 µM protein in 50 mM potassium phosphate buffer (pH 8.0), and normalized by subtracting the baseline recorded for the buffer under similar conditions. Temperature induced melting was evaluated by measurement of molar ellipticity (222 nm) at different temperatures that were increased to 90°C at a constant rate of 1°C min^−1^.

### Gas Chromatography

Gas chromatography was performed as described earlier [6], [22]. For enantioselectivity analysis of α-HCH, a chiral column Elite cyclodex B (30 m×0.25 mm×1 µm; Perkin Elmer) was used. Standard (+) and (−) enantiomers (Dr. Ehrenstorfer GmbH, Augsburg, Germany) were used for assigning the chirality. The temperature program used was 50°C for 2 min which was increased to 100°C at the rate of 20°C min^−1^, and further to 155°C at 0.5°C min^−1^, where it was held for 10 min. The injector and detector temperatures were fixed at 250 and 320°C, respectively. Under these conditions, the retention time of (+) and (−) enantiomers was 105.0 and 105.5 min, respectively.

### Generation of Mutant LinAs

Three sets of mutant LinAs were studied (**Table 3**). First set corresponds to the mutants M1–M4 that, compared to LinA-type1, carry eight (F68Y, C71T, L96C, F113Y, D115N, R129L, A131G & T133M), six (L96C, F113Y, D115N, R129L, A131G & T133M), five (F113Y, D115N, R129L, A131G & T133M) and three changes (R129L, A131G & T133M), respectively, in the C-terminal two-third region of the protein. The second set of mutants M5–M8, had seven (K20Q, A23G, F68Y, C71T, L96C, F113Y and D115N), five (K20Q, A23G, F68Y, C71T and L96C), four (K20Q, A23G, F68Y and C71T) and two changes (K20Q and A23G), respectively, in the N-terminal one-third region of the protein. The third set was of mutants M9 and M10 that had changes to K20Q and A23G, respectively. While genes for mutants M1, M3, M5 & M6 were obtained from the soil metagenome, discussed above (EU863886, EU863870, EU863854, EU863884 & EU863857, respectively), mutant M4 was constructed by ligating 157 bp Apa1 cut fragment of *linA*-type1with 314 bp ApaI cut fragment of mgA17R1 (EU863856). Similarly, mutants M8, M9 and M10 were constructed by ligating 157 bp ApaI fragment of mgA16R1 (EU863855), mgA1LR3 (EU863887) or mgA10R2 (EU863880), respectively, with 314 bp ApaI fragment of *linA*-type1. M7 was constructed by ligating 241 bp Tsp5091 cut fragment of mgA68R1 (EU863875) with 230 bp Tsp5091 fragment of -type1. The ligated products were amplified, cloned, and expressed following procedures discussed above. Activity and thermostability of all mutant LinA proteins was also determined as above.

### Crystallization of LinA-type2, Data Collection, and Structure Solution

Crystallization experiments of LinA-type2 were set up at 293 K by hanging-drop vapour diffusion method and using the sparse-matrix approach [23], [24]. Crystals were obtained in different preliminary conditions. The optimization process led to hexagonal shaped crystals (**Figure** **S1**) in conditions consisting of 2 µl of 12 mg ml^−1^ protein and 2 µl reservoir solution [0.1 M trisodium citrate dehydrate, pH 5.5 and 3% (w/v) PEG 3350 that reached a maximum size of about 0.8×0.3×0.3 mm in about 3 days. Crystals diffract weakly and after extensive tests, X-ray data were collected from a single crystal to 3.5 Å at room temperature using a MAR 345-dtb and a Rigaku micromax HF rotating-anode generator. The data were indexed, integrated and scaled using the MOSFLM [25] and SCALA [26], [27] programs. The unit-cell parameters of crystal were *a* = *b = *107.5; c = 52.3 Å with space group P6_3_22. The crystal mosaicity refined to ∼0.43 and an overall data completeness of 98.6% was obtained (**Table 4**). Calculation of the Matthews coefficient [28] suggested that the asymmetric unit most likely should contain about 7 subunits and corresponds to a solvent content of about 59.60% or a co-efficient value of 3.07.

Molecular replacement calculations using the structure of LinA-UT26 [8], were carried out using the Phaser [29] program as implemented in the CCP4 Package [27]. Initially searches identified 6 subunits with good packing and log-likelihood gain of 3436.46 using data between 30.72 to 3.5 Å. The subsequent examination of the difference Fourier maps led to the placement of the 7^th^ subunit. The model was initially subjected to rigid-body refinement, which led to R_factor_ of 29.8% and R_free_ 32.4% [30] respectively. After 10 cycles of restrained refinement with REFMAC [31] the R_factor_ decreased to 19.1% and R_free_ to 29.4%. Model building was carried out using the COOT program and the final values correspond to R_factor_ 17.8% and R_free_ 27.2% respectively. The refined model was validated with PROCHECK [32] (**Table 4**) and submitted to the Protein Data Bank (http://www.rcsb.org) (**Figure** **S3**). Structure analysis for hydrogen bonds, salt-bridges and other properties were carried out using the Protein interfaces, surfaces and assemblies server, PISA, at the European Bioinformatics Institute (http://www.ebi.ac.uk/pdbe/prot_int/pistart.html) [33].

### In Silico Docking

Docking calculations were carried out using GOLD suite 5.1 [34]. The (+) and (−) enantiomers of α-HCH were constructed and subjected to full energy minimization using Insight II 2000.1 Builder module (http://www.accelrys.com). The co-ordinates of the -type1 (PDB: 3A76) and the -type2 (present work) were used for docking the substrates. The complexes were evaluated using Goldscore. A maximum of 10 models were evaluated for each using the genetic algorithm method. Default GOLD parameters were used for other values in the calculations. The best predicted substrate-protein complex were analyzed.

### Accession Codes

The structure factors and co-ordinates have been deposited in the *Protein Data Bank* (http://www.rcsb.org) under the id 3S5C.

## Supporting Information

Figure S1 **Crystals of the LinA-type2.**
(PDF)

Figure S2 **2Fo-Fc density contoured at 1σ level depicted as a blue mesh around the R31’ and E81 residues in the LinA-type2 protein.** Other regions of the protein are depicted in cartoon representation.
(TIF)

Figure S3 **Protein Data Bank summary report for 3S5C.**
(PDF)
