# Supplementary figures and images for: Crystal Structure of the Hexachlorocyclohexane Dehydrochlorinase (LinA-Type2): Mutational Analysis, Thermostability and Enantioselectivity
Source: PLoS One. 2012 Nov 27;7(11):e50373. doi: 10.1371/journal.pone.0050373 (PMC3507683; doi:10.1371/journal.pone.0050373)

Figure S1. Crystals of LinA-type2 in the spacegroup  $P6_322$

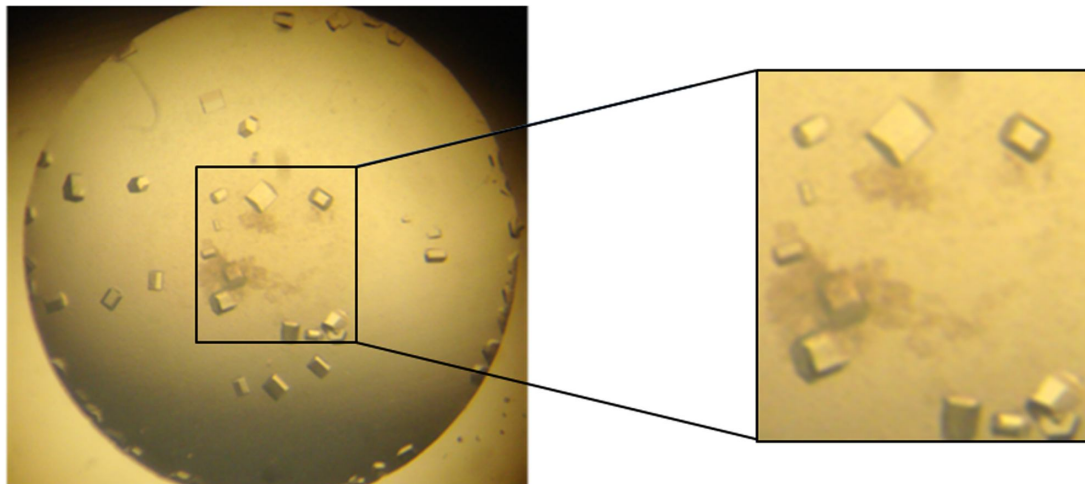

Supplement: Figure S1 — Crystals of the LinA-type2. (PDF) [file pone.0050373.s001.pdf]

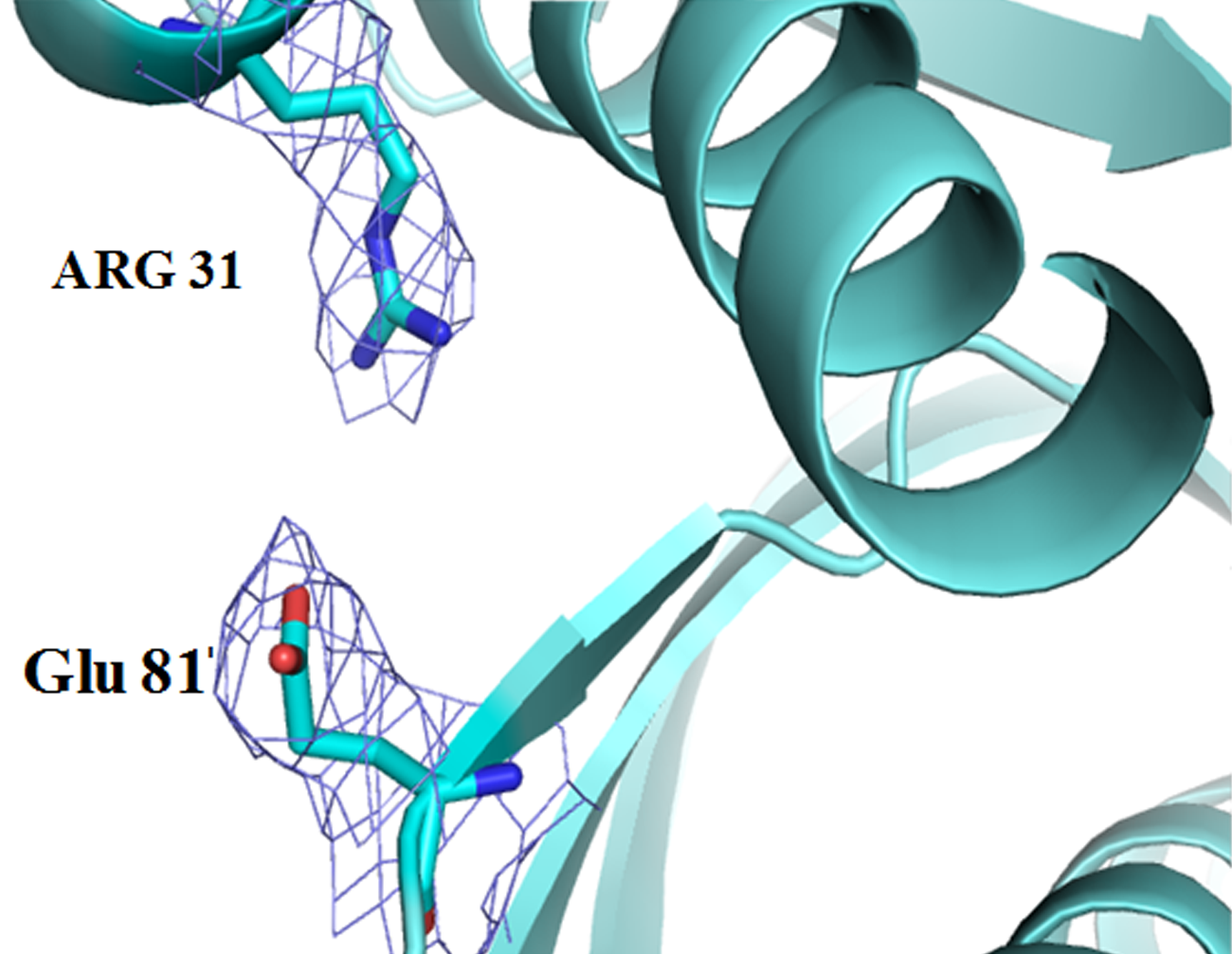

Supplement: Figure S2 — 2Fo-Fc density contoured at 1σ level depicted as a blue mesh around the R31’ and E81 residues in the LinA-type2 protein. Other regions of the protein are depicted in cartoon representation. (TIF) [file pone.0050373.s002.tif]
