# Supplementary material for: Crystal Structure of the Hexachlorocyclohexane Dehydrochlorinase (LinA-Type2): Mutational Analysis, Thermostability and Enantioselectivity
Source: PLoS One. 2012 Nov 27;7(11):e50373. doi: 10.1371/journal.pone.0050373 (PMC3507683; doi:10.1371/journal.pone.0050373)
Supplement: Figure S3 — Protein Data Bank summary report for 3S5C. (PDF) [file pone.0050373.s003.pdf]

CONFIDENTIAL VALIDATION REPORT

wwPDB Validation Report

PDB ID: 3S5C
RCSB ID: RCSB065780
TITLE: Crystal Structure of a Hexachlorocyclohexane dehydrochlorinase (LinA) from metagenome source
AUTHOR(S): R.Ramachandran, V.Kukshal, A.Macwan, A.Kumar

Summary of PDB Entry Validation

The results of the validation of this PDB entry are shown below. No major issues were raised during data processing.

Sequence Validation

The reported biological sequence shows no discrepancy with UniProt sequence (code B5ANU3).

The reported biological sequence and the sequence given in the coordinates show no discrepancy.

Biological Assembly

The biological assembly predicted by PISA is a trimer. This agrees with author's annotation.

Summary of Structure Factor Validation

Resolution	
High Resolution (Author reported)	3.50
High Resolution (Calculated by SFCHECK, V7.02.4)	3.50
High Resolution (Calculated by REFMAC, V5.5.0109)	3.500
Low Resolution (Author reported)	30.72
Low Resolution (Calculated by SFCHECK, V7.02.4)	30.72
Low Resolution (Calculated by REFMAC, V5.5.0109)	30.715

Crystal data	

Space group	P 63 2 2
Total number of reflections	18669
Number of reflections used	17700
Completeness of data	98.8

R-factors	
R-factor (Author reported)	0.179
R-factor (Calculated by SFCHECK, V7.02.4)	0.238
R-factor (Calculated by REFMAC, V5.5.0109)	0.1829
Free R-factor (Author reported)	0.271
Free R-factor (Calculated by SFCHECK, V7.02.4)	0.320
Free R-factor (Calculated by REFMAC, V5.5.0109)	0.2721

Structure quality	
Average Real space R-factor (Deviation) (Calculated by SFCHECK, V7.02.4)	0.1166
Average Real space R-factor (Deviation) (Calculated by MAPMAN, V7.8.5)	0.1899
Average Real-space correlation coefficient (Deviation) (Calculated by SFCHECK, V7.02.4)	0.9654
Average Real-space correlation coefficient (Deviation) (Calculated by MAPMAN, V7.8.5)	0.925
Average Occupancy-weighted avg temperature factor (Deviation)	50.19

Wilson statistics (PHENIX, V1.6-289)	
Wilson B-factor	58.36
Wilson Scale	0.62

Padilla-Yeates statistics for twin detection (PHENIX, V1.6-289)	
Padilla-Yeates <|L|>	0.440
Padilla-Yeates <L*L>	0.264

CONFIDENTIAL VALIDATION REPORT
